# News and Coming Events

**Published:** 1909-07-10

**Authors:** 


### News and Coming Events.

The London Hospital Medical College Endowment Fund has received a gift of £2,000 through Dr. Luther Martin, an old student of the hospital.

Mr. H. Morriston Davies, M.C. (Cantab.), F.R.C.S., has been appointed assistant surgeon to University College Hospital, Gower Street, W.C.

Dr. W. Wynn Westcott, J.P., coroner for North-East London, has recently been elected president of the Coroners' Society of England and Wales, and Mr. Walter Schröder, Deputy Coroner for Central London, has been reappointed honorary secretary of the society.

The retirement is announced from his post as Senior Surgeon to the Cancer Hospital, Fulham Road, of Mr. Frederick Bowreman Jessett, F.R.C.S. Mr. Jessett must surely be the *doyen* of the active members of London hospital staffs, but no one who has seen him operate would guess the full tale of years he has been in harness. For Mr. Jessett was already qualified before the Medical Council was entrusted with the compilation of the first Medical Register. His published works include many contributions to the literature of cancer, and he has been President of the British Gynæcological Society.

The late Mrs. Charlotte Greenhill, of Hyde Park, London, who died in April, left £162,737, of which a large amount, estimated at £90,000, is bequeathed to charities. Among the bequests are £2,000 to the Royal Free Hospital, Gray's Inn Road; £1,000 each to the Middlesex Hospital, the Children's Hospital, Paddington Green, and the West London Hospital, Hammersmith; and £200 each to the West London Hospital Ladies' Association and the Ladies' Association of the Royal Free Hospital. Subject to other legacies the testatrix left one-third of the residue of her estate to the Royal Free Hospital, Gray's Inn Road, and one-sixth each to the Children's Hospital, Paddington Green, and the Royal Ophthalmic Hospital, City Road.

Her Majesty the Queen has once more shown how deep and practical an interest she takes in the welfare of medical charities. By signifying her approval of the proposed endowment of a bed in the York County Hospital in memory of the late Canon Fleming, and by endorsing this with a personal donation, Her Majesty has given cause for much satisfaction to the many friends and admirers of the lamented Canon of York Minster, and to the managers and supporters of the hospital of that city.

The Prince and Princess of Wales, the Duke and Duchess of Connaught, and Princess Christian gave their patronage to the Carnival, held on July 1, at the Crystal Palace, in aid of the fund for the removal of King's College Hospital to South London. A feature of the Carnival was a bazaar, and the presidents of stalls included the Premier's wife, Lady Lansdowne, and many other distinguished ladies. Dramatic performances took place at intervals, and many well-known music-hall and concert artists assisted in the three variety performances. In the grounds athletic sports were organised by the Blackheath Harriers, and the programme also included a cricket match, gipsy encampment, pastoral plays, band performances, wrestling matches, swimming races, and an ascent of Professor Huntington's balloon.

The Chelsea Hospital for Women has received from Lady Ilchester and the members of the Ladies' Committee the sum of £260, being the proceeds of the concert recently given at Holland House by kind permission of Mary, Countess of Ilchester.

His Majesty the King during his recent visit to Gloucester made the following gratifying reference to the local Infirmary :— " I am happy to take this opportunity to inform you that I give my permission for the title 'Royal' to be prefixed henceforward to the name of the Gloucester General Infirmary in recognition of the admirable work done by those responsible for its management, and the devoted and successful efforts of its staff in the prevention and alleviation of human suffering."

The annual general meeting of the British Medical Association for the present year will be held in the Assembly Hall, Belfast, on Friday, July 23, at 12 noon. This meeting is to comply with Article XII. of the Rules of the Association, and will adjourn forthwith until Tuesday, July 27, at 2.30 o'clock. The annual representative meeting will be held in the Assembly Hall, Belfast, on July 23 (and following days as required), immediately after the annual general meeting, fixed for 12 noon on Friday, July 23.

The late Dr. Hall, of St. John's Wood, and formerly of Crouch End, left estate of the net value of £87,679. His many charitable bequests include legacies to Epsom College, the British Medical Benevolent Fund, the National Hospital for the Paralysed and Epileptic, and the Royal London Ophthalmic Hospital, and £1,000 to the Research Defence Society. The balance of Dr. Hall's estate goes to the University of Glasgow, of which he was a graduate, for the foundation of tutorial fellowships.

The financial position of the Great Northern Central Hospital, Holloway, is causing grave anxiety to the committee of management. The income for the year 1908 showed a serious falling off, especially in donations and legacies, with the result that there was a deficiency between receipts and expenditure at the end of the year of £4,600. This deficiency was met by recourse to a loan from the bankers, raising the amount owing to them to £10,800. The receipts for the year show little or no improvement. At the present moment £3,000 is owing to tradesmen, and there are practically no funds available.

The death is announced at Hampstead, at the age of 58, of Mr. Frederick Nutcombe Hume, M.R.C.S.Eng., medical superintendent of the North-Western Fever Hospital. Mr. Hume was a major of the R.A.M.C. (Territorial), and had served as a surgeon to the National Aid Society's ambulance in the Servo-Turkish war in 1876; and in 1885 he saw active service again during the Servo-Bulgarian war, while a year later he acted as surgeon in the Russo-Turkish campaign. He was also successively surgeon and director to the English Hospital at Belgrade. In recognition of his various military services, Mr. Hume was decorated with the Takova, Medjidieh, and St. Sava Orders, and was awarded Servian and Turkish war medals.

The opening ceremony of the new Manchester Royal Infirmary took place on Tuesday, July 6, when the buildings were declared open by His Majesty the King, who was accompanied by Queen Alexandra.

---

We learn that, previous to the opening of tuberculosis exhibitions in various centres in England, the exhibits shown at the recent Tuberculosis Exhibition at Whitechapel have been transferred to the "White City" at Shepherd's Bush.

---

At a meeting of the Physiological Society held at Oxford on June 26, the president, Professor F. Gotch, presented to Dr. F. W. Pavy, in commemoration of his eightieth birthday, a silver bowl inscribed as follows: "Frederick William Pavy, M.D., F.R.S., May 29th, 1909. From the Physiological Society in token of affection and admiration."

---

Mr. Sinclair White, F.R.C.S., on the occasion of the last meeting of the Central Council, was unanimously elected President of the British Medical Association in place of the late Mr. Simeon Snell. Mr. Sinclair White is Senior Surgeon to the Royal Infirmary, Sheffield, and was largely responsible for the success of the last annual meeting of the association which was held in that city.

---

The Duke and Duchess of Connaught visited University College on Thursday, July 1, when His Royal Highness opened the Hogarth Fair, which has been organised by the students with the object of raising funds to pay off the debt on their new athletic ground at Perivale. Their Royal Highnesses were received by Lord Reay, Vice-Chairman of the College Council, and Lord Herschell. In the quadrangle was mounted a guard of honour of the University College and Hospital Company of the Officers' Training Corps, who received the visitors with a Royal salute and were afterwards inspected by His Royal Highness.

---

A conference, under the auspices of the Invalid Children's Aid Association, was held at Denison House, on Tuesday and Wednesday, June 22 and 23. The Earl of Aberdeen presided, and spoke of the health work done in Ireland. Speeches were made by Mr. Warrington Howard and by the delegates from Glasgow, Liverpool, Manchester, etc., while Miss Adler contributed a paper on "Open-air Schools." At the afternoon session, presided over by Dr. Chittenden Bridges and subsequently by Dr. Arthur Saunders, some account was given by Miss Broadbent of phthisical cases referred to the association, and papers were read on the "Anti-tuberculosis Dispensary System," by Dr. D. J. Williamson; on the "Results of Treatment of Tuberculosis in the Metropolitan Asylums Board Homes for Children," by Dr. Elliot Browne; and on the "Children's Sanatorium at Holt," by Dr. Gillam. A reception was given in the evening by Sir Alfred and Lady Fripp, while on Wednesday morning, when the conference was resumed, the chair was taken by the Duchess of Sutherland and later by Mrs. Burgwin, of the L.C.C. At the afternoon session Dr. Loch presided, and the subject was the Poor Law Report with regard to medical aid for children. After Dr. Loch's paper Dr. Allan spoke on health societies, and was followed by Dr. Lowrie and others. On both Tuesday and Wednesday the children's section of the Tuberculosis Exhibition, recently held in Whitechapel, was shown to the delegates in a room of Denison House.

---

The sixty-eighth annual meeting of the Medico-Psychological Association of Great Britain and Ireland will be opened on the morning of Thursday, July 22, at the West Riding Asylum, Wakefield.

---

The annual general meeting of the Asylum Workers' Association will be held on Tuesday, July 13, at 3 p.m., at the Medical Society's House, 11 Chandos Street, Cavendish Square, W., under the presidency of Sir William Collins, M.D., F.R.C.S., M.P.

---

An announcement appears in the *London Gazette*, dated at the Lord Chamberlain's Office, St. James's Palace, July 6, to the effect that the King has been graciously pleased to appoint Sir Alan Reeve Manby, M.V.O., M.D., to be Physician Extraordinary to his Majesty.

---

The annual luncheon of the Continental Anglo-American Medical Society will be held on Thursday, July 29, at Belfast, during the annual meeting of the British Medical Association. Members intending to be present should communicate with Dr. C. G. Jarvis, the hon. secretary, 81, Boulevard Malesherbes, Paris.

---

His Majesty's permission has been granted to Mr. Edward S. Crispin, M.R.C.S., Assistant Director of the Soudan Medical Department, Khartoum, to accept and to wear the Imperial Ottoman Order of the Osmanieh of the Fourth Class, conferred upon him by the Khedive of Egypt in recognition of valuable services.

---

Professor Ernest W. White, M.B. (Lond.), has been elected Emeritus Professor of King's College, London (University of London), on his retirement, after twenty years' service, from the professorship of psychological medicine. The vacancy thus occasioned at King's College has been filled by the appointment to the professorship of psychological medicine of Dr. Robert Hunter Steen, M.D. (Lond.).

---

The many medical men who have been associated with the National Hospital in Queen Square, London, will be interested to learn of the recent presentation to Mr. Edward Hill, Senior Dispenser to the National Hospital for the Paralysed and Epileptic, on his retirement after twenty-two years' service. The gift took the form of a solid silver tea service and a tray suitably engraved.

---

Voting took place on July 1 at the Royal College of Surgeons of England for the election of three Fellows to the Council of the College. The President, Sir Henry Morris, announced the result of the poll to be as follows: Sir Watson Cheyne, 420 votes; Mr. William Harrison Cripps, 385 votes; Mr. Richard Clement Lucas, 356 votes; Mr. John Bland-Sutton, 333 votes; Mr. Walter Hylton Jessop, 328 votes; Mr. Charles Alfred Ballance, 261 votes; Mr. Arthur Mayo Robson, 208 votes. The President thereupon declared Sir Watson Cheyne, Mr. Lucas, and Mr. Cripps to be elected members of the Council. The poll was the largest on record, 971 Fellows recording their votes. Mr. H. J. Price and Mr. Haig Brodie were the scrutineers. All three successful candidates have already served upon the Council of the College; but Mr. Harrison Cripps, who was elected in the first instance to fill an intermediate vacancy, had held office for less than the full period.

